# Effect of particle size on phytochemical composition and antioxidant properties of *Sargassum cristaefolium* ethanol extract

**DOI:** 10.1038/s41598-021-95769-y

**Published:** 2021-09-09

**Authors:** E. S. Prasedya, A. Frediansyah, N. W. R. Martyasari, B. K. Ilhami, A. S. Abidin, H. Padmi, Fahrurrozi, A. B. Juanssilfero, S. Widyastuti, A. L. Sunarwidhi

**Affiliations:** 1 Bioscience and Biotechnology Research Centre, Faculty of Mathematics and Natural Sciences, University of Mataram, Mataram, Indonesia; 2 Department of Biology, Faculty of Mathematics and Natural Sciences, University of Mataram, Mataram, Indonesia; 3 Research Division for Natural Product Technology (BPTBA), Indonesian Institute of Sciences (LIPI), Wonosari, Indonesia; 4 Pharmaceutical Institute, University of Tuebingen, Tuebingen, Germany; 5 Laboratory of Biochemical Engineering and Food Biotechnology, Research Centre for Biotechnology, Indonesian Institute of Sciences (LIPI), Bogor, Indonesia; 6 Faculty of Food Technology and Agroindustry, University of Mataram, Mataram, Indonesia; 7 Department of Pharmacy, Medical Faculty, University of Mataram, Mataram, Indonesia

**Keywords:** Biochemistry, Biological techniques, Chemical biology, Drug discovery, Medical research

## Abstract

Sample particle size is an important parameter in the solid–liquid extraction system of natural products for obtaining their bioactive compounds. This study evaluates the effect of sample particle size on the phytochemical composition and antioxidant activity of brown macroalgae *Sargassum cristaefolium*. The crude ethanol extract was extracted from dried powders of *S.cristeafolium* with various particle sizes (> 4000 µm, > 250 µm, > 125 µm, > 45 µm, and < 45 µm). The ethanolic extracts of *S.cristaefolium* were analysed for Total Phenolic Content (TPC), Total Flavonoid Content (TFC), phenolic compound concentration and antioxidant activities. The extract yield and phytochemical composition were more abundant in smaller particle sizes. Furthermore, the TPC (14.19 ± 2.08 mg GAE/g extract to 43.27 ± 2.56 mg GAE/g extract) and TFC (9.6 ± 1.8 mg QE/g extract to 70.27 ± 3.59 mg QE/g extract) values also significantly increased as particle sizes decreased. In addition, phenolic compounds epicatechin (EC), epicatechin gallate (ECG), epigallocatechin (EGC), and Epigallocatechin gallate (EGCG) concentration were frequently increased in samples of smaller particle sizes based on two-way ANOVA and Tukey’s multiple comparison analysis. These results correlate with the significantly stronger antioxidant activity in samples with smaller particle sizes. The smallest particle size (< 45 µm) demonstrated the strongest antioxidant activity based on DPPH, ABTS, hydroxyl assay and FRAP. In addition, ramp function graph evaluates the desired particle size for maximum phytochemical composition and antioxidant activity is 44 µm. In conclusion, current results show the importance of particle size reduction of macroalgae samples to increase the effectivity of its biological activity.

## Introduction

Seaweeds or also referred to as macroalgae are currently being explored for sources of novel and sustainable compounds for both pharmaceutical and nutraceutical purposes^1^. Biologically active substances known as “bioactive compounds” have been well reported in various natural resources, including seaweeds^2–4^. Bioactive compounds such as carotenoids, polyphenols, tocotrienols, sulfated polysaccharides are of high interest^5^. Due to this vast variety of bioactive constituents, seaweeds have been proven to demonstrate various biological activities, especially antioxidant activity by inhibiting reactive oxygen radical-mediated oxidative stress.

Marine macroalgae belong to three major classes or phyla: Chlorophyceae (green algae), Rhodophyceae (red algae), and Phaeophyceae (brown algae). The most extensively studied macroalgae are those of the Phaeophyceae family^6^. Brown macroalgae are well known to be rich in polyphenol compounds which potentially contribute to its antioxidant capacity^7^. The brown seaweed *Sargassum* species have shown strong antioxidant activity^8^. The brown macroalgae *Sargassum cristaefolium* is one of the most abundant *Sargassum* species in the coastal area of Lombok, Indonesia.

Several parameters have been reported to influence the amount and composition of the potential bioactive compounds in extracts^9^. Furthermore, these parameters also affect the biological activity of the extract. Some of them include the extraction solvent, temperature, extraction time, storage conditions, and also sample particle size^10^. The phytochemical compositions of extracts have been reported to be influenced by the particle size of the extracted sample. Previous reports have demonstrated that reduction of sample particle size has a significant effect on the amount of bioactive constituents obtained^11^. However, this is the first report to evaluate the effect of particle size on the phytochemical composition and antioxidant activity of macroalgae. Due to the increasing demand to obtain bioactive compounds from macroalgae, there is a need to understand every aspect during the extraction process of the macroalgae biomass especially the particle size as it is the very initial step. Thus, this paper aims to evaluate the impact of particle size on the extraction yield, phenol content, and antioxidant activity of brown macroalgae *S.cristaefolium*.

## Materials and methods

### Chemicals and standard solutions

All chemicals were of analytical grade. Ethanol were from Merck (Darmstadt, Germany) and l-ascorbic acid by Sigma-Aldrich (Steinhein, Germany). Water used for all experiments and solutions was obtained from Milli-Q water purification system (Millipore) (Bedford, MA, USA). Folin-Ciocalteu’s phenol reagent was purchased from Merck (Darmstadt, Germany). DPPH and ABTS reagents were purchased from Tokyo Chemical Industry Co. Ltd (TCI). Standards of catechin (C), epicatechin (EC), epicatechin gallate (ECG), epigallocatechin (EGC), Quercetin (QUE), Procatechuic acid (PCA), and Epigallocatechin gallate (EGCG) were from Sigma-Aldrich (St. Louis, MO, USA).

### Macroalgae sample collection and preparation

Samples of *Sargassum cristaefolium* were harvested offshore from Lendang luar beach, West Lombok, Indonesia (8.459692, 116.029639) in February 2021. The macroalgae samples were identified based on the algae online database^12^. The samples were washed with seawater and cleaned from sand debris before transported to the Laboratorium. Approximately 200 g of fresh *S.cristaefolium* samples were left to dry at room temperature (24 °C) regulated with an air conditioner for 4 days until the dry biomass reaches constant weight (± 50 g dry weight). The Fungicide tablets 1% concentration (Rely + On, Virkom) were also applied to avoid contamination from fungal growth. Dried samples were then stored in ziplock bags with silica gels until further use.

### Macroalgae sample partition and extraction

Dried macroalgae samples were grounded with a food-grade grain miller to decrease the particle size. The ground samples were sieved on a Sieve shaker (Retsch As 200, USA) to separate the samples into various sizes; < 45 µm, > 45 µm, > 125 µm, > 250 µm, and > 4000 µm. All the dried samples with different particle sizes were prepared before subjected to ethanol extraction. The extraction process was conducted via cold maceration with the sample to solvent ratio of 1:10. The samples were stirred vigorously in ethanol solvent for 30 min and then subjected to centrifugation at 8000 rpm for 10 min (Tomy MX-307, Tomy Seiko Co., Ltd, Japan). The supernatant was collected and filtered with a filter cloth. This step was repeated 3 times, the final filtrate was then evaporated with a rotary evaporator at 45 °C and 50 rpm (Rotavapor R-215, Switzerland). Obtained extract pastes were then stored at 4 °C until further use.

#### Determination of total phenolic content

The Total Phenolic Contents (TPC) of the *S.cristaefolium* different particle sizes were determined by Folin-Ciocalteu colorimetric method as described by Ainsworth and Gillespie with some minor modifications^13^. Gallic acid (GAE) solution was used as standard and prepared by dissolving 10 mg in 10 mL of ethanol (1 mg/mL). Various concentrations of GAE (10–500 µg/mL) were prepared from the stock solution. Approximately 100 µL of the sample (1 mg/mL) was combined and mixed with 0.75 mL of the Folin-Ciocalteu reagent (diluted tenfold in dH_2_O before use). The liquid mixture was incubated at room temperature for 5 min. The mixture was then added about 750 µL sodium carbonate (Na_2_CO_3_), the mixture was mixed gently with pipetting. After 90 min, the absorbance of the mixture was measured at 725 nm with a UV–Vis spectrophotometer (Multiskan-Go, Thermo Scientific). The TPC value of samples was revealed as Gallic acid equivalents in milligrams per 100 g of the extract.

#### Determination of total flavonoid content

The total flavonoid content was measured by a colorimetric assay. About 100 µL of the sample was added to 4 mL of dH_2_O. Then followed by the addition of 300 µL of 5% sodium nitrite. After 5 min, 300 µL of 10% aluminium chloride was added. The mixture was incubated for an additional 6 min before the addition of 2 mL 1 M sodium hydroxide. Immediately, the mixture was diluted by the addition of 3.3 mL dH_2_O and vortexed. The absorbance was determined at 510 nm versus a blank. Quercetin was used as the standard for the calibration curve. The total flavonoid content of the sample was expressed as mg quercetin equivalents per gram of sample (mg/g).

### High resolution mass spectromety analysis

For spectrometry analysis, a Q Exactive™ High-Resolution Accurate Mass LC–MS/MS (Thermo Scientific™) attached to a Thermo Scientific™ VanquishTM Flex UHPLC system was used. Using a flow rate of 0.3 mL/min and a 5 μL injection volume, the HPLC method was (0.1 percent formic acid in H_2_O MS grade as solvent A and 0.1 percent formic acid in Acetonitrile MS grade as solvent B), a gradient of 5 percent to 90 percent B in 16 min, an isocratic of 90 percent B for 4 min, and an additional 5 min 90 percent to 5 percent B. The separation was carried out on a 2.6 m Accucore™ Phenyl Hexyl 100 × 2 mm column, with an MS acquisition range of 150 to 1800 m/z. We used a sheath gas flow rate of 15, an auxiliary gas flow rate of 5, a spray voltage of 3.6 kV, a capillary temperature of 320 °C, an auxiliary gas heater temperature of 30 °C, and an S-lens RF level of 50. The resolution was set to 70,000 for the entire MS, with an AGC target of 3e6 and a maximum IT of 250 ms. Additionally, the resolution for dd-MS^2^ was set to 17,500, with an AGC target of 1e5 and a maximum IT of 60 ms. Furthermore, the loop count was set to 5, and the (N) CE/steeped nce was 18, 35, 53, with TopN and isolation window set to 5 and 1.0 mz, respectively. For *dd* setting, the minimum AGC target was 9e3, with an intensity of 1.3e5 and a charge exclusion of 4–8, > 8. The exclude isotope must be enabled, and the dynamic exclusion time must be set to 10 s. Caffeine was used as a calibrant in the study.

### High performance liquid chromatography (HPLC) analysis

The concentrations of phenolic compounds were analyzed using high-performance chromatography (HPLC) (LC-20AB, SPD- M20A photodiode array detector (PDA), Shimazu, Kyoto Japan) equipped with an InfinityLab Poroshell 120 EC-C18 chromatography column, 150 mm length, 4.6 mm width, and particle size 2.7 μm at column oven temperature 26 °C. The binary gradient method was used in HPLC analysis incorporated 2% acetic acid dissolved in water (A) and a mixture of concentrated acetic acid, water, and acetonitrile (1:9:40 v/v/v) (B). The total runtime of the analysis was 93 min referring to the method described elsewhere^14^ as follows: (a) initially 0–25 min, 10–30% B; (b) 25–50 min, 30–40% B; (c) 50–75 min, 40–90% B; (d) 75–93 min, 10% B. An amount of 20 μL of the samples was injected onto the column, and three wavelengths 280, 360 and 520 nm were chosen for analysis in this investigation using HPLC–DAD. For quantitative purposes, a calibration curve was constructed by analysis of known concentrations of different standard compounds.

### DPPH radical scavenging assay

Radical scavenging activity against DPPH radical was measured as described by Mansour et al.^15^, with minor modifications. A sample of 100 µL with various concentrations (10–4000 µg/mL) was mixed with 100 µL solution of DPPH in a 96-well plate. A mixture of the sample with solvent without DPPH radical was added for blank. The plate was allowed to stand in the dark (RT) for 20 min. The absorbance was measured at 520 nm. The sample radical scavenging activity was measured with the equation below:

1 $$ {\text{Scavenging}}\;{\text{effect}}\;(\% ) = \left[ {1 - \frac{{\left( {{\text{Abs}}_{{{\text{sample}}}} - {\text{Abs}}_{{{\text{blank}}}} } \right)}}{{{\text{Abs}}_{{{\text{control}}}} }}} \right] \times 100\% $$

### ABTS radical scavenging assay

The scavenging activity of extracts was measured against ABTS radical cation according to the method of Nenandis et al.^16^, with minor modifications. The stock solutions prepared included 7 mM ABTS aqueous solution and a 2.4 mM potassium persulfate solution. The working solution was prepared by mixing the two stock solutions in equal quantities and allowing them to react for 16 h at room temperature in the dark. The solution was then diluted by mixing 250 µL ABTS with 12 mL ethanol to obtain an absorbance of around 0.700 units at 734 nm using a spectrophotometer. Fresh ABTS solution was prepared for each assay. A volume of 1 mL sample extracts of various concentrations (10–4000 µg/mL) was mixed with 1 mL ABTS solution and the absorbance was measured at 734 nm after 7 min of incubation at room temperature. The ABTS scavenging activity of the extracts was calculated with the same equation above (1).

### Hydroxial anti radical scavenging assay

The hydroxyl radical scavenging ability of the extracts was evaluated based on a Fenton-type reaction^17^. A volume of 1 mL sample in various concentrations (10–4000 µg/mL) was mixed with 1 mL of 9 mM ferric sulfate, 1 mL of 9 mM ethanolic salicylic acid, and 1 mL of 9 mM hydrogen peroxide. The mixture was incubated for 30 min at 37 °C. The absorbance was measured at 510 nm. Ascorbic acid was used as the positive control. The activity was calculated as Eq. (1).

### Ferric reducing antioxidant power assay (FRAPS)

The Ferrous Reducing Antioxidant Power Assay (FRAPS) of the extracts were evaluated by the method described by Mansoori et al.^18^ with minor modifications. The Fe^2+^ can be monitored by measuring the formation of Perl’s Prussian blue at 700 nm. A volume of 0.25 mL samples/standard at different concentrations (10–4000 µg/mL) were mixed with 0.625 mL potassium buffer (0.2 M) and 0.625 mL of 1% potassium ferricyanide [K_3_Fe(CN)_6_] solution were added into the test tubes. The reaction mixtures were incubated for 20 min at 50 °C to complete the reaction. To this mixture, a volume of 0.625 mL 10% trichloroacetic acid (TCA) solution was added. The total mixture was centrifuged at 3000 rpm for 10 min. The supernatant (1.8 mL) was collected and mixed with 1.8 mL dH_2_O and 0.36 mL 0.1% Ferric chloride (FeCl_3_) solution. The absorbance of the solution was recorded at 700 nm using a spectrophotometer. Increased absorbance of the reaction mixture indicates increased reducing capacity.

### Statistical analysis

All assays were carried out in triplicates. Data were presented as mean ± SD to evaluate significant relationships between experimental parameters. The statistical ANOVA analysis with Tukey’s multiple comparisons. The IC_50_ value and graphs were generated with Graphpad Prism software (v9.1.10). Optimal particle size was predicted using Response Surface Method in Design Expert 13.

## Results and discussion

### Effect of particle size on extract yield

**Figure 1:**
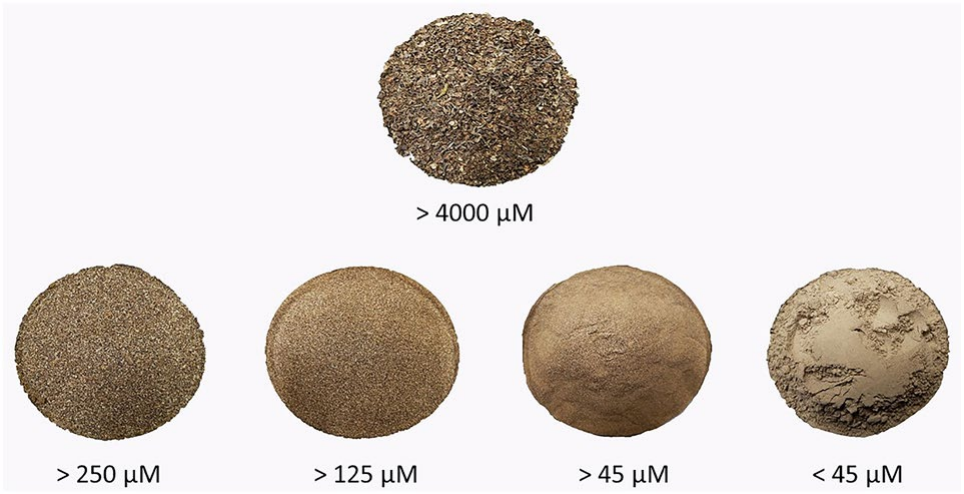
Brown macroalgae *S.cristaefolium* and their corresponding thallus grounded to different particle sizes.

**Figure 2:**
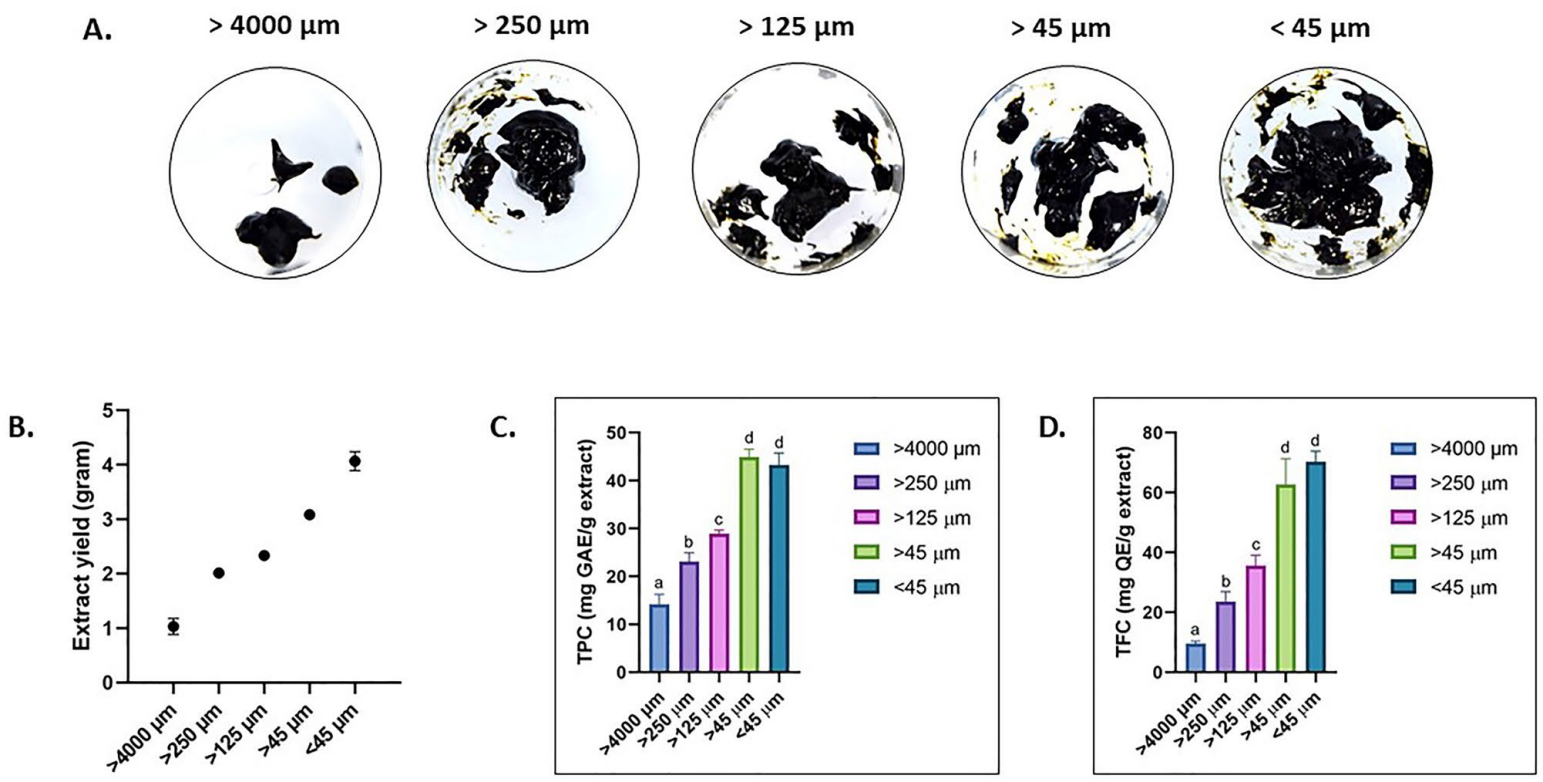
Effect of particle size on *S.cristaefolium* extract (**A**) total yield, (**B**) yield increase, (**C**) TPC-Total Phenolic Content, and (**D**) TFC-Total Flavonoid Content. Different letters indicating significant difference (*p* < 0.05).

Prior to extraction, the samples were separated into various sizes (< 45 µm, > 45 µm, > 125 µm, > 250 µm, and > 4000 µm) (Fig. 1). The fraction of < 45 µm showed the maximum extract yield with approximately 4% of algal powder dry weight (Fig. 2A). A Previous study reported that extraction of green tea leaves from smaller particle sizes produced higher extract yield (> 1000 µm)^19,20^. In the case of macroalgae samples, a study on red macroalgae *Laminaria* spp. shown that smaller particle sizes produced higher extract yield^21^. Overall, the total extract yield increased as the sample particle size decreases (Fig. 2B). By reducing the particle size of the algal powder, the contact surface area of the sample and the ethanol solvent used for extraction is increased^22^. Thus resulting in a significantly higher amount of extract yield obtained.

### Effect of particle size on total phenolic content (TPC) and total flavonoid content (TFC)

The phytochemical composition of *S.cristaefolium* ethanol extracts shown higher contents in samples of smaller particle sizes (Table 1). The extract from samples with smaller particle sizes produced significantly higher Total Phenolic Content (TPC) and Total Flavonoid Content (TFC) (Fig. 2C,D). However, no significant differences were observed between particle sizes of > 45 µm and < 45 µm. Numerous reports have demonstrated the importance of sample particle size in the solid–liquid extraction system^23^. However, in some studies reducing particle size does not always contribute to higher bioactive compounds retrieved. In tea, the reduction in particle size led to a higher decrease in almost all phenolic compound catechins measured^24^. In addition, in some cases, the granulometric characteristics do not influence the amount of bioactive compounds extracted^25^. For example, the total phenolic content and antioxidant activity of green propolis were increased regardless of the particle size. But in the case of macroalgae or seaweed, reduction of particle size is necessary due to its rigid biomass texture. The size reduction of a biological sample before extraction maximizes the surface area, which in turn enhances the transfer of bioactive compounds from the biological material to the solvent^26^. The content of TPC and TFC from several *Sargassum* species had been demonstrated in other studies^27^. However, due to different parameters and sample preparation, the results are sometimes incomparable. Based on our results, we suggest that smaller particle sizes in macroalgae dried sample is beneficial for the improvement of the TPC and TFC content which has significant effects on its antioxidant activity.

**Table 1:** Phytochemical composition and antioxidant activity of different sample particle size of *S.cristaefolium* ethanol extract.

	> 4000 µm	> 250 µm	> 125 µm	> 45 µm	< 45 µm	Ascorbic acid
Yield (%)	1.03 ± 0.15^a^	2.05 ± 0.4^b^	2.33 ± 0.7^c^	3.08 ± 0.08^d^	4.07 ± 0.18^e^	
**Phytochemical composition**						
Total Phenolic content (mg GAE/g extract)	14.19 ± 2.08^a^	23.14 ± 1.86^b^	28.92 ± 1.78^c^	44.95 ± 2.62^d^	43.27 ± 2.56^d^	
Total Flavonoid content (mg QE/g extract)	9.6 ± 1.8^a^	23.67 ± 3.25^b^	35.67 ± 3.41^c^	62.73 ± 8.61^d^	70.27 ± 3.59^d^	
**Free radical scavenging activity IC50 (µg/mL)**						
DPPH radical scavenging activity (IC_50_)	803.1 ± 1.89^a^	822.4 ± 1.78^a^	438.1 ± 1.32^b^	254.9 ± 2.5^c^	202.7 ± 1.22^c^	37.94 ± 2.89^d^
ABTS radical scavenging activity (IC_50_)	758.7 ± 1.48^a^	772.4 ± 3.78^a^	321.5 ± 2.13^b^	170.1 ± 3.26^c^	151.5 ± 2.12^c^	24.53 ± 2.54^d^
Hydroxyl radical scavenging activity (IC_50_)	2163 ± 2.96^a^	1840 ± 2.24^b^	1310 ± 3.32^c^	1068 ± 2.51^d^	930.8 ± 4.14^d^	33.11 ± 1.61^e^
**Ion reducing activity**						
Ferric reducing power (Abs at 700 nm)	0.574 ± 0.013^a^	0.602 ± 0.01^a^	0.759 ± 0.029^b^	0.944 ± 0.015^c^	0.993 ± 0.04^c^	1.523 ± 0.06^d^

Different letters indicating significant difference between columns (p < 0.05).

### Effect of particle size on metabolite profle (HR-ESI–MS analysis)

**Figure 3:**
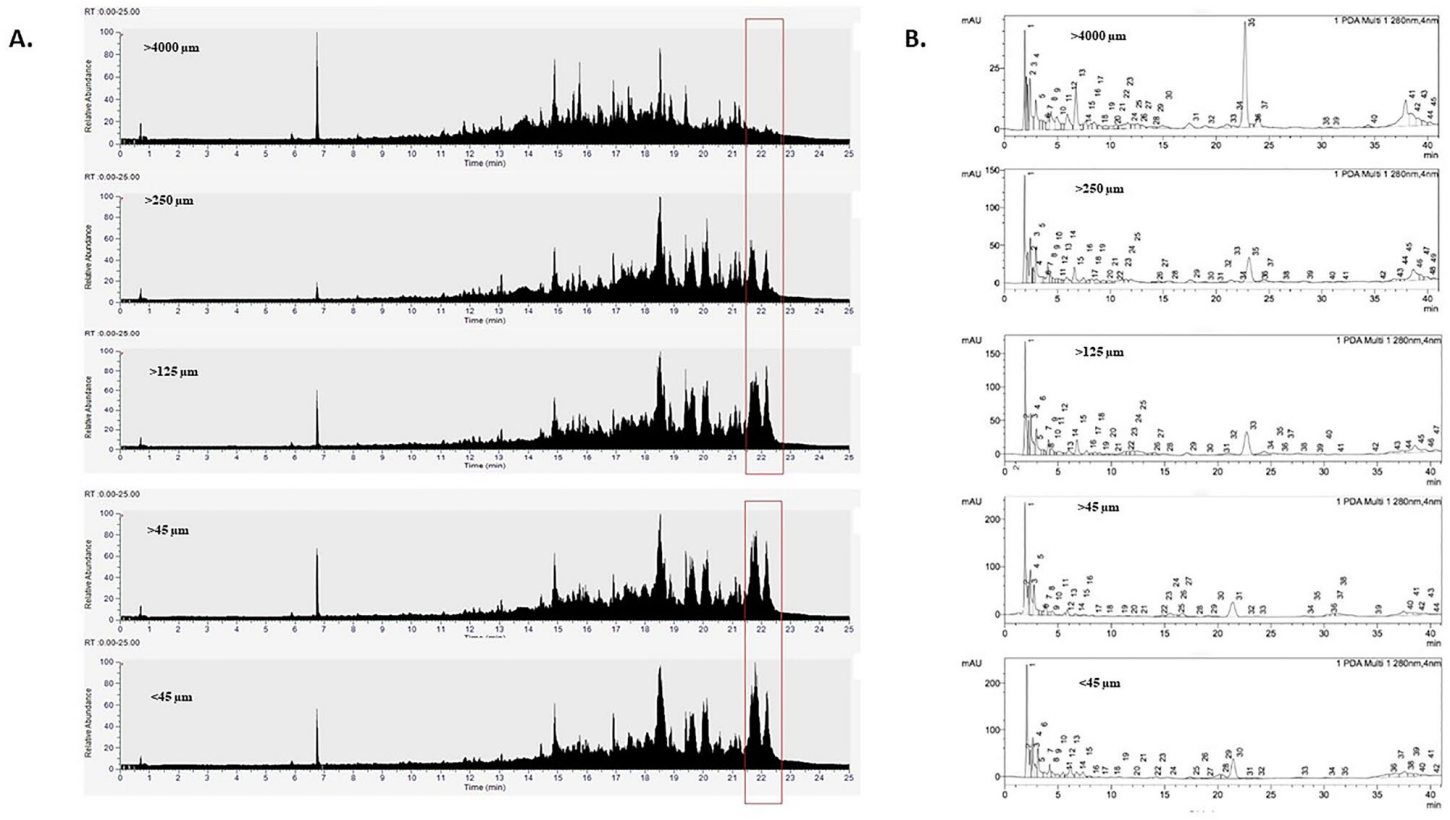
Mass Spectrometry analysis of *S. cristaefolium* extracts with varying particle sizes. (**A**) Comparative HR-ESI–MS analysis showing different metabolite compositions. The red box indicates changes in phytochemical composition. (**B**) HPLC analysis of phenolic compounds showing increased concentration in samples with smaller particles. Known standards are listed as follows, 1. EGC-Epigallocatechin; 2. PCA-Protocatechuic Acid; 3. C-Catechin hydrate; 4. ECG-Epicatechin gallate; 5. EGCG-Epigallocatechin gallate; 6. QUE-Quercetin.

Particle size is one parameter that has a significant effect on the extraction of bioactive compounds from natural products^28,29^. Our results show that there is increased metabolite composition based on reduction of sample particle size (Fig. 3A). Interestingly in samples with particle size larger than 4000 µm, a significant reduction of non-polar metabolites was observed in the 22 min range. Previous research also increase in bioactive compound composition in smaller particle size of various plant leaves^30^. The reduction of particle size not only increases the diffusivity of bioactive compounds, but also helps to rupture the cell walls of the sample^31,32^. The reduction of sample particle size important in macroalgae samples, as the cell walls in macroalgae are extremely thick^33^.

### Effect of particle size on phenolic profile (HPLC analysis)

In order to evaluate the effect of particle size on the phenolic profile of *S.cristaefolium* extract, the HPLC analysis was performed (Fig. 3B). This HPLC analysis revealed the presence of some major phenolic compounds such as EGC-Epigallocatechin; PCA-Protocatechuic Acid; C-Catechin hydrate; ECG-Epicatechin gallate; EGCG-Epigallocatechin gallate; QUE-Quercetin (Table 2). Presence of phenolic compounds have been well reported in seaweeds. Tabled results showed that epicatechin, which was the most frequently appearing phenolic compound in *S.cristaefolium* extract. These group of polyphenols are also proven to be abundant in other seaweeds such as *Undaria pinnatifida* and the red seaweed *Palmaria palmata*^34^. Based on our results the phenolic compound EGC, ECG, EGCG were significantly higher in samples with smaller particle sizes. Other similar studies of HPLC analysis of phenolic compounds in algal samples showed that EGC could range from 0.13 to 3.8 mg/g or from 0.25 to 0.76 mg/g^35,36^. The immense difference of these values could caused by many factors, not only the species and origin but also extraction process.

**Table 2:** Amounts (mg/g sample) of selected phenolic compounds (*EGC* epigallocatechin; *PCA* protocatechuic acid; *C* catechin hydrate; *ECG* epicatechin gallate; *EGCG* epigallocatechin gallate; *QUE* quercetin) in *S.cristaefolium* extracts.

Phenolic compound	Concentration (mg/g)				
	> 4000 µm	> 250 µm	> 125 µm	> 45 µm	< 45 µm
EGC	3.30 ± 0.26^a^	11.48 ± 0.4^b^	13.79 ± 0.68^c^	19.73 ± 0.76^d^	20.04 ± 0.5^e^
PCA	0.010 ± 0.003^a^	0.014 ± 0.004^a^	0.018 ± 0.004^a^	0.020 ± 0.005^a^	0.023 ± 0.002^a^
C	0.069 ± 0.003^a^	0.074 ± 0.006^a^	0.082 ± 0.006^a^	0.087 ± 0.005^a^	0.091 ± 0.004^a^
ECG	4.70 ± 0.17^a^	12.01 ± 0.48^b^	13. 99 ± 0.58^c^	20.67 ± 0.89^d^	24.97 ± 0.78^e^
EGCG	4.13 ± 0.08^a^	6.10 ± 0.57^b^	7.33 ± 0.71^c^	9.25 ± 0.50^d^	10.67 ± 0.21^e^
QUE	0.51 ± 0.04^a^	0.57 ± 0.03^a^	0.61 ± 0.09^a^	0.84 ± 0.11^a^	1.20 ± 0.08^a^

Different letters indicating significant difference between columns (p < 0.05).

### Effect of particle size in antioxidant activity

The role of particle size has been previously reported to significantly affect the antioxidant activity of the extract. This has been observed in several natural products including wheat, banana, and blackberries^37–39^. As previously described in our results, the phytochemical composition are significantly affected by sample particle size. A significant correlation has been reported between the antioxidant activity of natural products due to their phenolic constituents^40–42^.

**Figure 4:**
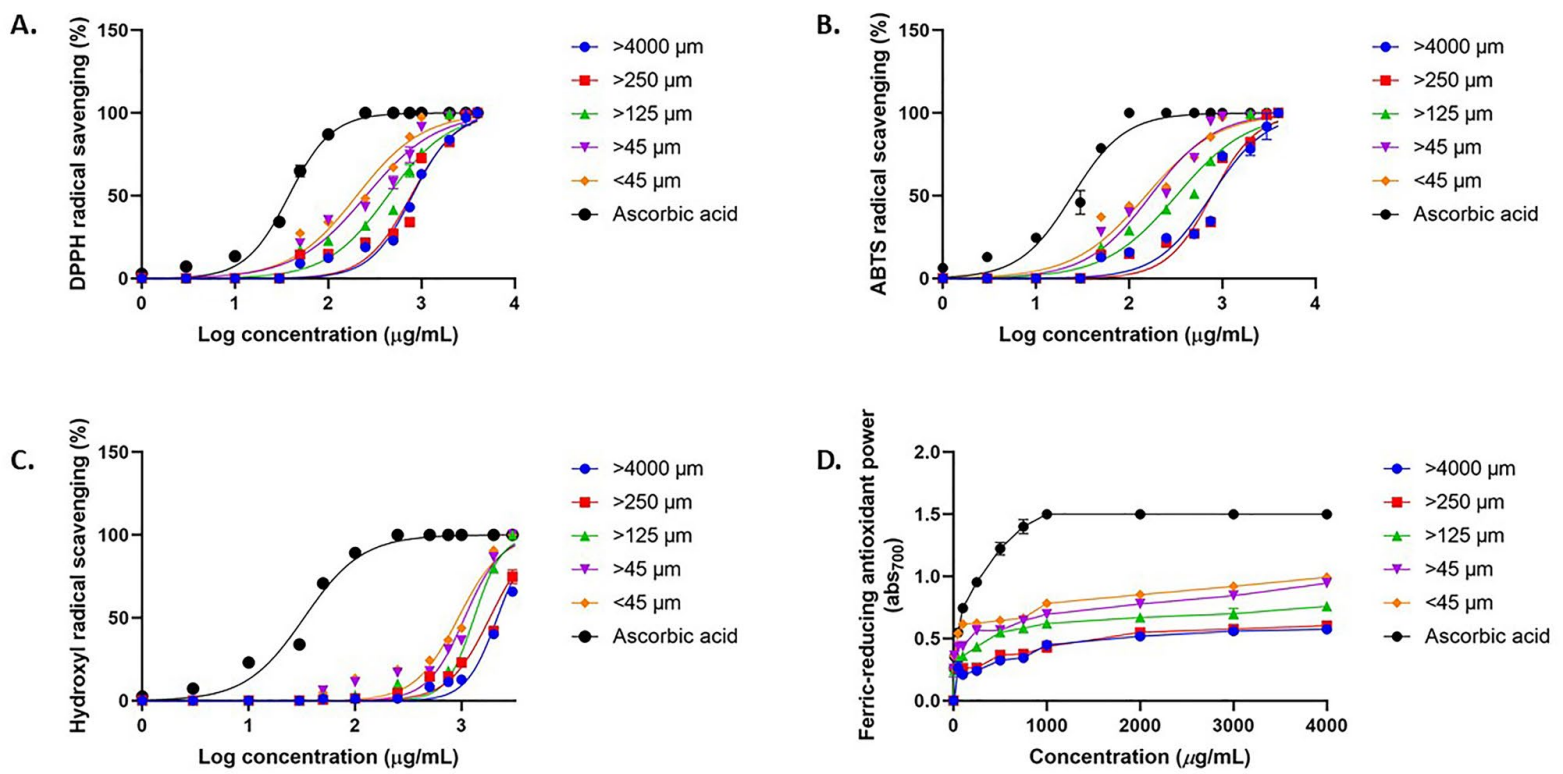
Effect of particle size on *S.cristaefolium* extract antioxidant activity based on (**A**) DPPH, (**B**) ABTS, (**C**) hydroxyl, and (**D**) ferric reducing assay. Experiments are done in triplicates, values are expressed as means ± SEM.

The antioxidant activity of macroalgae has been well reported in previous studies^2,38,43,44^. The most important and studied bioactive compounds from marine macroalgae are polyphenols, polysaccharides, carotenoids, and polyunstaturated fatty acids^45,46^. The phenolic compounds are the main contributors to the antioxidant activity, and this is shown that *S.cristaefolium* extract with higher TPC value and phenolic compound concentrations show stronger antioxidant activity. The extract from the smaller particle size samples produced stronger antioxidant activity based on DPPH, ABTS, hydroxyl assay, including Ferric Reducing Antioxidant Power (FRAP) (Fig. 4). Brown macroalgae *Sargassum wightii* has shown strong inhibition against free-radical DPPH and ABTS with IC_50_ around 320 to 800 µg/mL^47^. Another study shown presence of major antioxidant components such as Sargahydroquinoic acid (SHQA), sargachromanol (SCM) and sargaquinoic acid (SQA) in *Sargassum serratifolium.* These components demonstrate strong antioxidant capacities with IC_50_ values below 100 µg/mL^48^. Among all antioxidant assay, *S.cristaefolium* extract show strongest activity against ABTS radical. The ABTS assay is considered more sensitive in identifying antioxidant activity because of the faster reaction kinetics, and its response to antioxidants is higher compared to other radicals^49^. Overall, the extract with smaller particle size has more potential to react with free radicals and terminate them into non-reactive stable form.

### Validation analysis of optimum particle size

**Figure 5:**
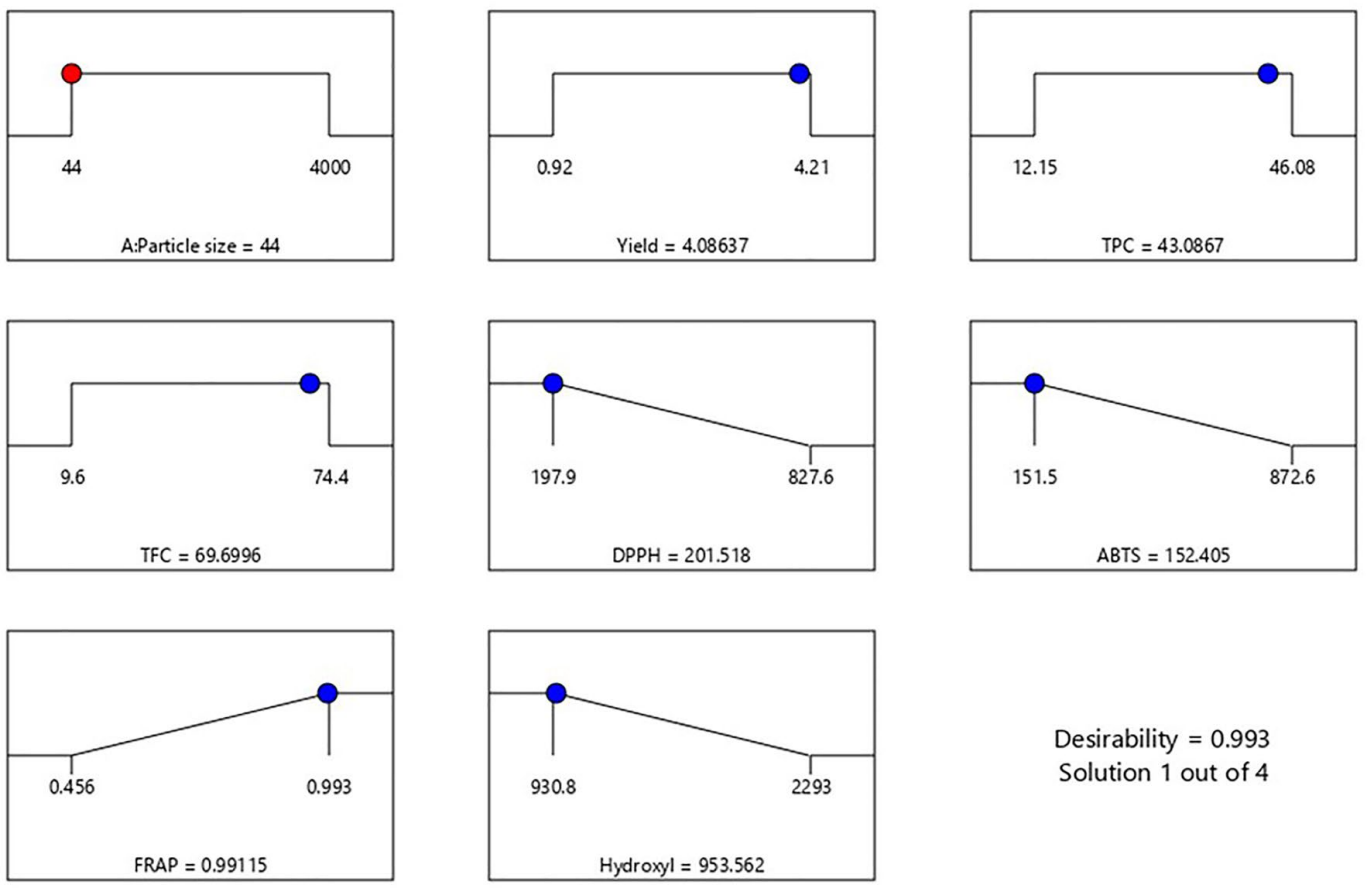
Desirability analysis. Plots ramp showing the optimal particle size that maximize TPC, TFC, and antioxidant activity.

Desirability optimization of sample particle size condition was carried out for accumulative phytochemical composition and antioxidant activity. During desirability determination, the criteria proposed for selecting the optimum particle size conditions for macroalgae *S.cristaefolium* were for independent responses; TPC and TFC were maximized while Antioxidant activities (DPPH, ABTS, Hydroxyl anti-radical scavenging assay, FRAPS) were minimized. By applying the desirability function approach, the optimum particle size of *S.cristaefolium* samples was obtained. Figure 5 showed desirability ramps that were developed from optimum points via numerical optimization. A triplicate experiment was set up to validate the optimized condition. The composite desirability (0.993) is close to 1, which indicates the settings seem to achieve favourable results for all responses as a whole^50^. Based on predictive model, the optimum particle size is 44 µm to obtain favourable results of phytochemical composition and antioxidant activity. This prediction correlates to results showing that sample particle sizes < 45 µm showed maximum phytochemical composition and antioxidant activity.

## Conclusion

The present study has shown that particle size is important in improving the phytochemical constituents and antioxidant activity of macroalgae *S.cristaefolium*. The constituent of bioactive compounds; phenolic and flavonoid compounds increased due to reduction of particle size. Metabolite profile and major phenolic compounds were also significantly increased compared to larger particles. Furthermore, this contributes to the increase of antioxidant activity of the extracts with smaller particle size. Particle size less than 45 µm showed the best phytochemical composition and antioxidant activity. This procedure may be extended to other applications to obtain more valuable bioactive constituents from macroalgae samples.
